# Comparison of Diagnostic Performance of Semi-Quantitative Knee Ultrasound and Knee Radiography with MRI: Oulu Knee Osteoarthritis Study

**DOI:** 10.1038/srep22365

**Published:** 2016-03-01

**Authors:** Jana Podlipská, Ali Guermazi, Petri Lehenkari, Jaakko Niinimäki, Frank W. Roemer, Jari P. Arokoski, Päivi Kaukinen, Esa Liukkonen, Eveliina Lammentausta, Miika T. Nieminen, Osmo Tervonen, Juhani M. Koski, Simo Saarakkala

**Affiliations:** 1 Research Unit of Medical Imaging, Physics and Technology, Infotech Oulu, University of Oulu, Oulu, Finland; 2 Department of Radiology, Boston University School of Medicine, Boston, MA, USA; 3 Departments of Anatomy and Surgery Clinic, Medical Research Center, University of Oulu and Oulu University Hospital, Oulu, Finland; 4 Research Unit of Medical Imaging, Physics and Technology, Medical Research Center, University of Oulu and Oulu University Hospital, Oulu, Finland; 5 Department of Diagnostic Radiology, Oulu University Hospital, Oulu, Finland; 6 Institute of Clinical Medicine, University of Eastern Finland, Kuopio, Finland; 7 Department of Physical and Rehabilitation Medicine, Kuopio University Hospital, Kuopio, Finland; 8 Department of Radiology, University of Erlangen-Nuremberg, Erlangen, Germany; 9 Department of Internal Medicine, Mikkeli Central Hospital, Mikkeli, Finland

## Abstract

Osteoarthritis (OA) is a common degenerative musculoskeletal disease highly prevalent in aging societies worldwide. Traditionally, knee OA is diagnosed using conventional radiography. However, structural changes of articular cartilage or menisci cannot be directly evaluated using this method. On the other hand, ultrasound is a promising tool able to provide direct information on soft tissue degeneration. The aim of our study was to systematically determine the site-specific diagnostic performance of semi-quantitative ultrasound grading of knee femoral articular cartilage, osteophytes and meniscal extrusion, and of radiographic assessment of joint space narrowing and osteophytes, using MRI as a reference standard. Eighty asymptomatic and 79 symptomatic subjects with mean age of 57.7 years were included in the study. Ultrasound performed best in the assessment of femoral medial and lateral osteophytes, and medial meniscal extrusion. In comparison to radiography, ultrasound performed better or at least equally well in identification of tibio-femoral osteophytes, medial meniscal extrusion and medial femoral cartilage morphological degeneration. Ultrasound provides relevant additional diagnostic information on tissue-specific morphological changes not depicted by conventional radiography. Consequently, the use of ultrasound as a complementary imaging tool along with radiography may enable more accurate and cost-effective diagnostics of knee osteoarthritis at the primary healthcare level.

Osteoarthritis (OA) is a common musculoskeletal degenerative disease. Prevalence of knee OA in aging populations is increasing worldwide leading to reduced quality of life and working disability which has major implications for healthcare and overall economy^1^,^2^. OA is no longer seen as a disease of “wear and tear” but rather conceptualized as a whole-organ disorder^3^. Besides articular cartilage degeneration, formation of osteophytes, bone erosion, meniscus atrophy, effusion and synovial inflammation are structural and compositional hallmarks of the disease. In the past few years, the role of diagnostic imaging has increased in detection, prognosis and follow up of the individual features in OA^4^.

In clinical practice, severity of knee OA is primarily assessed using conventional radiography especially by evaluation of joint space narrowing (JSN) and to some extent by the Kellgren-Lawrence (KL) grading^5^. However, structural alterations visible on radiographs such as bone abnormalities and JSN are known to appear only at relatively late stages of the disease^6^. In contrast to conventional KL grading, which is a composite score combining osteophyte presence and JSN for the whole knee, feature-oriented atlas-based compartmental Osteoarthritis Research Society International (OARSI) radiographic grading is becoming more frequently deployed in clinical research^7^. Although little direct information on soft tissue degeneration is revealed by radiography, and some studies reported insensitivity to progression of cartilage thinning^8^, JSN is still being widely applied as an indirect indicator of tibio-femoral cartilage loss. However, it is also known that JSN is a surrogate of both cartilage thinning and meniscal extrusion, and there are no means to directly evaluate cartilage and meniscus morphological damage from radiographs^8^,^9^,^10^,^11^.

To date, magnetic resonance imaging (MRI) is considered the most accurate imaging modality in the assessment of knee OA^4^. Semi-quantitative whole-joint scoring systems have been developed, validated and successfully used in several OA studies to evaluate multi-feature morphological degeneration within the knee joint^12^. Despite its high sensitivity, MRI is not usually used as an initial imaging technique for knee OA due to practical and cost reasons.

Recently, high-resolution ultrasound has become of great interest in knee OA research^13^,^14^,^15^,^16^,^17^,^18^,^19^,^20^,^21^,^22^. Morphological changes in bone, meniscus and femoral cartilage can be reliably depicted and semi-quantitatively and/or quantitatively assessed as single features^13^,^14^,^15^,^16^,^17^,^18^. Evidence on ultrasound validity in comparison to traditional OA imaging modalities is increasing^13^,^14^,^15^,^17^,^23^,^24^,^25^,^26^,^27^. Naredo *et al.* reported that ultrasound findings, such as medial meniscal extrusion, are related to knee pain and radiographic medial JSN^27^. Ultrasound also correlates strongly with KL grading in the evaluation of morphological changes, although potential superiority or inferiority of ultrasound over radiography has not been confirmed^13^. Over 10 years ago, Tarhan *et al.*^23^ already demonstrated significant agreement of ultrasound with MRI in the assessment of femoral cartilage and soft tissue deterioration^23^. Consequently, increasing evidence in the scientific literature supports the idea of deploying ultrasound as one of the first-line modalities for detection of morphological changes in knee OA. However, systematic feature- and site-specific cross-comparison of ultrasound, radiography and MRI is still missing in the current literature.

The aim of our study was to systematically determine the site-specific diagnostic performance of ultrasound for semi-quantitative grading of femoral articular cartilage, osteophytes and meniscal extrusion, and of radiographic assessment of JSN and osteophytes, using MRI as a reference tool.

## Methods

Our retrospective study is part of the Oulu Knee Osteoarthritis (OKOA) study including 80 symptomatic and 80 asymptomatic subjects. Consecutive recruitment of participants was carried out between October 2012 and April 2014. Written informed consent was obtained from each participant. The study was carried out in accordance with the Declaration of Helsinki and approved by the Ethical Committee of Northern Ostrobothnia Hospital District, Oulu University Hospital (number 108/2010).

### Symptomatic group

**Figure 1:**
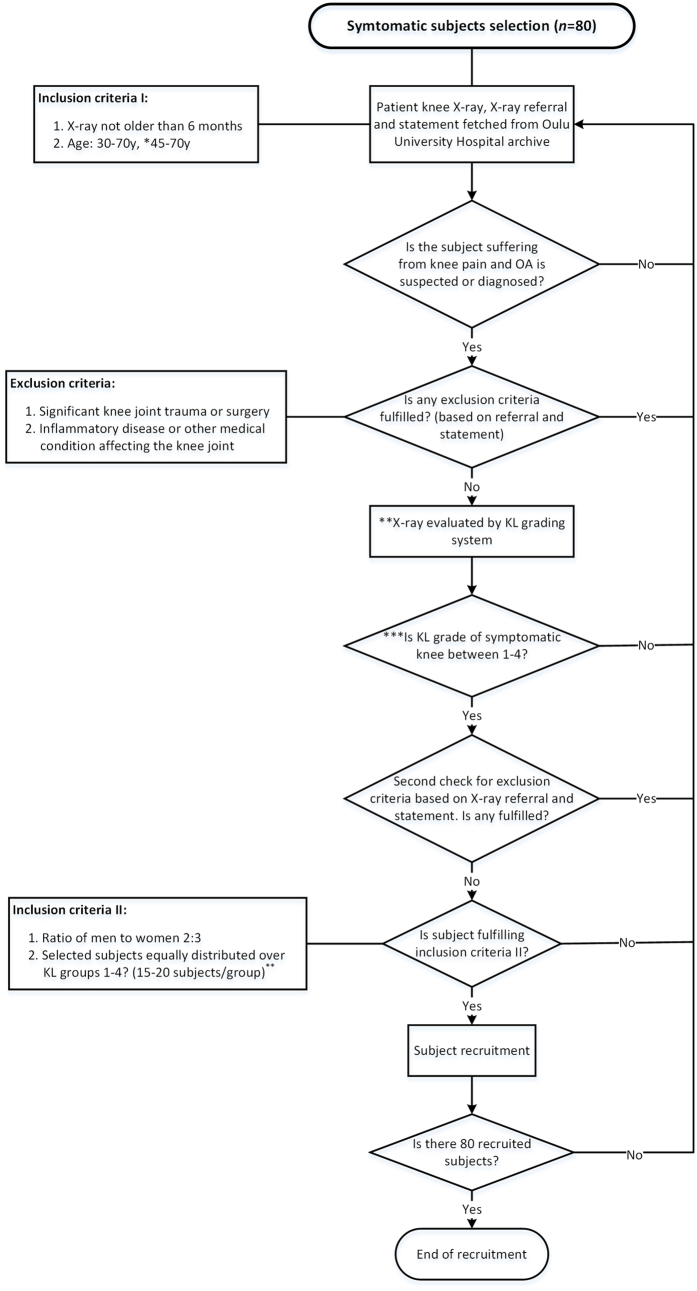
Flowchart describing the selection of symptomatic subjects. *After pilot examinations of 21 subjects the age range was modified to easier recognize the OA subjects (as OA prevalence increases with age). **X-rays were evaluated by a rheumatologist with 25 years of experience in reading knee X-rays. ***Except of subjects with KL 0 included in pilot examinations.

Eighty symptomatic subjects were selected from OA patients who were referred to either knee radiography due to non-specific knee pain, or referred to total knee arthroplasty (with radiographs available) at the Oulu University Hospital and Oulu municipality health centers. The subject selection is described in Fig. 1.

### Asymptomatic group

**Figure 2:**
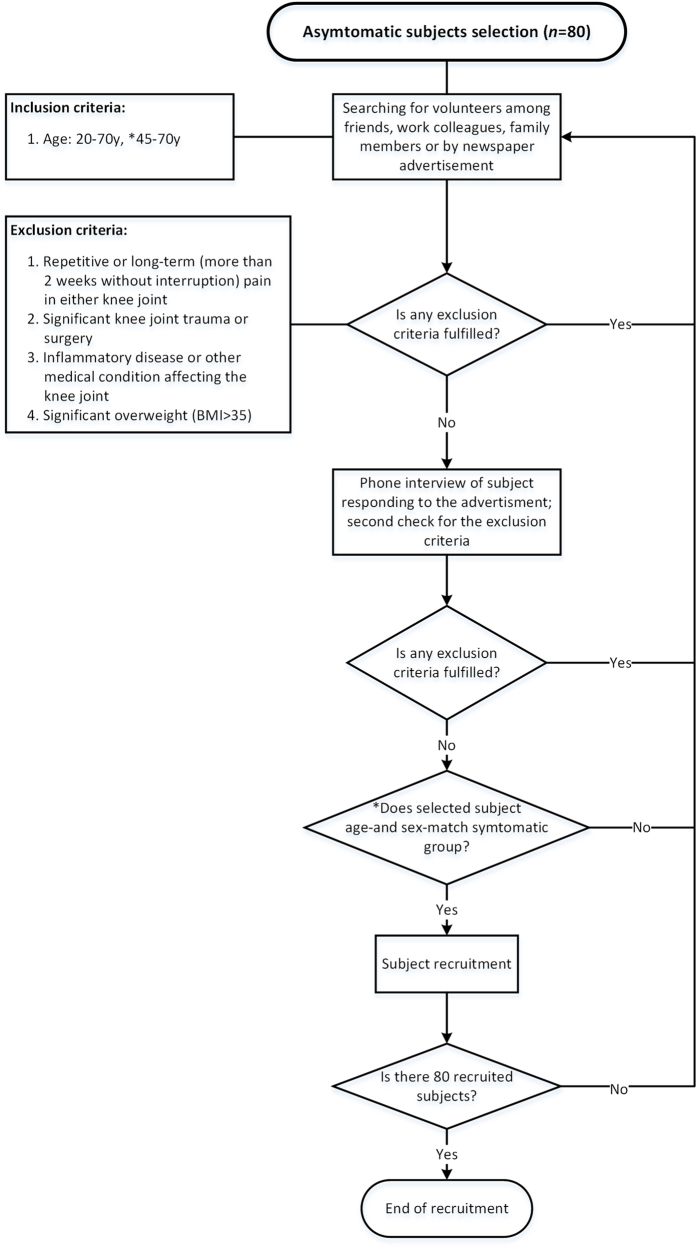
Flowchart describing the selection of asymptomatic subjects. *After pilot examinations of 25 subjects the age range was modified to age-match the symptomatic group.

Eighty asymptomatic subjects were selected from work colleagues, friends and by advertisement in local newspaper. Details of the recruitment process are described in Fig. 2.

### Radiography

Symptomatic subjects underwent bilateral weight-bearing postero-anterior radiography maximum of 6 months before ultrasound and MRI examinations. The X-ray beam was 10° caudally angulated and the knee was supported by a frame in 20° flexion and foot in 5° external rotation. The symptomatic knee was evaluated semi-quantitatively (grade 0–3) for medial and lateral JSN, and osteophytes (grade 0–3) in the medial and lateral femur and tibia by two readers experienced in radiographic evaluation, musculoskeletal radiologist (JN, 11 years of experience in radiographic evaluation) and orthopaedic surgeon (PL, 8 years of experience in radiographic evaluation), using the revised OARSI atlas^7^. Both readers were blinded to clinical, MRI, ultrasound and prior radiographic findings. Inter-reader reliability was evaluated and discrepancies were adjudicated in a separate session by the same readers. Final grade agreed by both readers was used in the diagnostic performance analysis.

### Ultrasound imaging

Dynamic ultrasound imaging was conducted using clinical ultrasound (LOGIQ E9, GE Healthcare, Milwaukee, WI, USA) with 15 MHz linear transducer ML6–15. B-mode imaging settings were kept constant for each subject and focus was always set at the level of region of interest. Knee ultrasound was performed by a trained sonographer (JP, undergoing three separate 2-day training sessions). The symptomatic knee was imaged in the patient group and knee of the dominant hand side was imaged in the asymptomatic group. Medial, sulcus and lateral site of femoral articular cartilage was depicted by constant speed proximal-distal transducer sweeping over the supra-patellar region as described by Saarakkala *et al.*^14^. Subsequently, dynamic anterior-posterior longitudinal scans from medial and lateral side of the extended knee were obtained for evaluation of osteophytes and meniscus integrity. Two video files were saved for each site.

Systematic semi-quantitative ultrasound femoral cartilage grading^14^ was performed by a rheumatologist (JMK, 25 years of experience in musculoskeletal ultrasound). He was blinded to subject grouping, clinical, radiographic and MRI findings. The presence and size of osteophytes was evaluated in medial-femoral, medial-tibial, lateral-femoral and lateral-tibial bone margin as follows: Grade 0 = no osteophyte present, Grade 1 = marginal osteophyte, Grade 2 = medium osteophyte and Grade 3 = large osteophyte^15^. Meniscal extrusion was measured as a perpendicular distance (mm) between the most distant meniscus border and line connecting the femoral and tibial bone ends (leading below osteophytes if present) (see Supplementary Fig. S1).

Ultrasound videos of 25 asymptomatic and 26 symptomatic subjects were randomly selected for the intra-reader reliability evaluation. The data were presented to the reader in random order 3 months after the first assessment.

### Magnetic resonance imaging

On the same day when ultrasound was performed, the knees were imaged with 3T MRI scanner (Siemens Skyra, Siemens Healthcare, Erlangen, Germany) using a 15-channel transmit/receive knee coil. Following sequences were carried out: sagittal T2-weighted dual-echo steady-state, 3D sagittal proton-density (PD) weighted SPACE fat-suppressed turbo spin-echo (TSE), coronal PD-weighted TSE and coronal T1-weighted TSE. Technical details of sequences can be found in Supplementary Table S1.

A musculoskeletal radiologist (AG, 15 years of experience in semi-quantitative MRI analysis of knee OA) who was blinded to subject grouping, clinical and other imaging findings systematically evaluated the MRI for structural changes of femoral and tibial cartilage, presence and size of medial and lateral osteophytes in femur and tibia, and extrusion of medial and lateral meniscus using the MRI Osteoarthritis Knee Score (MOAKS)^12^.

**Figure 3:**
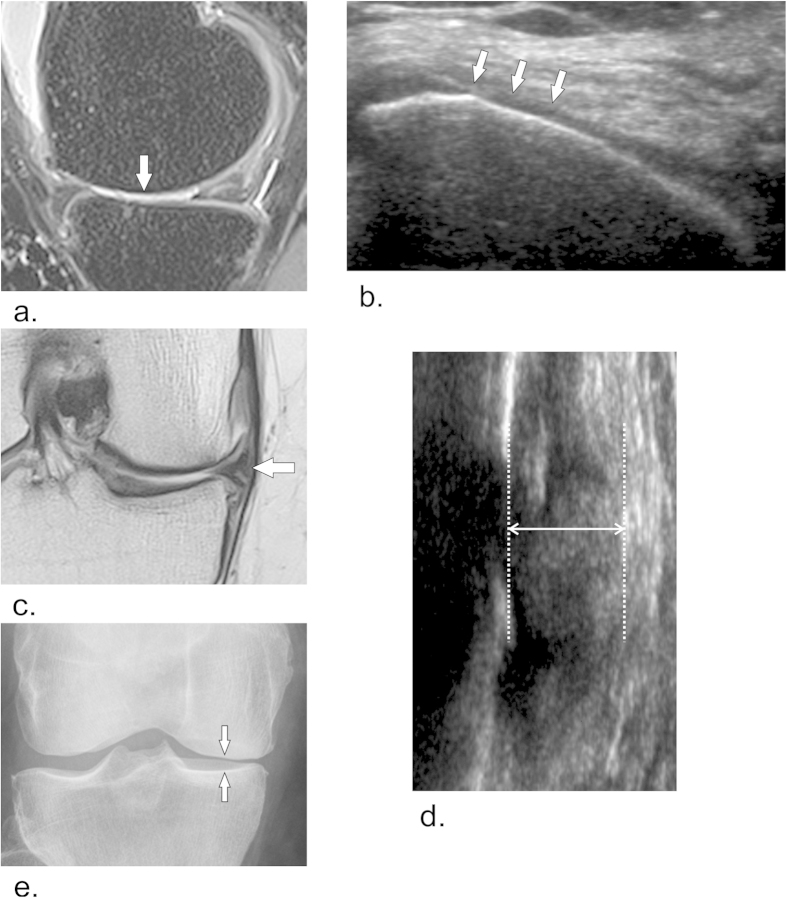
Example of structural cartilage changes and meniscal extrusion as seen in magnetic resonance images (MRI), ultrasound images and radiograph of 57-year-old symptomatic woman. Medial femoral condyle cartilage thinning indicated by white arrows in sagittal proton density weighted fat-suppressed MRI (**a**) and ultrasound transversal B-mode image (**b**). Medial meniscal extrusion can be observed in coronal proton density weighted MRI (**c**) (white arrow) and longitudinal B-mode ultrasound image (**d**) (double headed arrow). Anterior-posterior radiograph (**e**) demonstrates medial joint space narrowing (white arrows) as a surrogate of meniscal and cartilage structural changes.

Figure 3 shows an example of the same knee visualized by all three modalities. Cartilage structural degeneration and meniscal extrusion can be clearly distinguished by MRI and ultrasound images but not by radiography.

### Statistical analysis

Original ultrasound cartilage grade 1 combines evaluation of structural cartilage deterioration visible as a loss of interface sharpness and internal echogenicity variation reflecting the compositional alteration. On the other hand, MOAKS assesses only morphological changes. Therefore, ultrasound cartilage grade 1 was combined with grade 0 (morphologically intact) in our study. Both MOAKS cartilage grades, assessing any cartilage surface loss (Femoral cartilage I) and full thickness cartilage loss (Femoral cartilage II), were applied in the analysis. Ultrasound is able to depict the entire anterior and a significant proportion of central cartilage sub-regions^28^,^29^,^30^. In order to compare MOAKS and ultrasound grade of corresponding anatomical cartilage regions, the maximum from anterior and central sub-regional MOAKS grades was considered in medial and lateral femoral condyle. Likewise the maximum ultrasound grade from medial and sulcus region was used as ultrasound medial cartilage grade. We hypothesized that JSN is predominantly affected by cartilage loss in weight-bearing region and, therefore, we used maximum MOAKS grade of central femoral and central tibial sub-regions (Femoral-tibial central cartilage I and II) as a reference in individual condyle.

Linearly weighted Cohen’s kappa coefficient (κ_w_), percentage of exact agreement (PEA), percentage of close agreement (PCA, defined as a difference of ±1 between the readings/readers) and intra-class correlation coefficient (ICC) were calculated as appropriate to assess the intra-rater reliability of ultrasound grading and inter-rater reliability of radiographic OARSI grading.

The area under the receiver-operating characteristics (ROC) curve (AUC), sensitivity, specificity, accuracy, positive predictive value (PPV) and negative predictive value (NPV) with 95% confidence interval (CI) were calculated to assess the feature-specific diagnostic ability of ultrasound and radiography. Continuous distance measure of meniscal extrusion was used to calculate the AUC. To assess remaining diagnostic performance values a cutoff value of 3 mm was used to define pathological meniscal extrusion in ultrasound. It is known that JSN of only medial or lateral compartment paradoxically widens the complementary joint space^31^,^32^. To overcome this fact, we selected knees only involved by medial or symmetrical JSN to compare with MOAKS cartilage and meniscal extrusion grades in medial compartment applying the following criteria: medial JSN ≥ lateral JSN. Similarly we used criteria for lateral compartment: lateral JSN ≥ medial JSN. AUC lower than 0.7 was considered as indicator of poor performance, from 0.7 to 0.8 as fair, from 0.8 to 0.9 as good and greater than 0.9 as excellent performance. Statistical differences between ultrasound and radiographic ROC curves were investigated using the method of DeLong *et al.*^33^. Statistically significant differences were determined by *P-value* less than 0.05.

The statistical analyses were carried out using IBM SPSS software (ver. 20, SPSS Inc., Chicago, IL, USA), custom Microsoft Excel script and MedCalc software (ver. 15.6, MedCalc Software bvba, Belgium).

## Results

### Participants

Altogether 159 subjects were enrolled in our study. One symptomatic subject was excluded due to missing ultrasound video of sulcus cartilage. The detailed demographic characteristics of symptomatic and asymptomatic participants are listed in Table 1. The groups significantly differed (*P* < 0.001) in weight/BMI and knee flexion.

### Reliability

The intra-rater agreement for assessment of individual ultrasound features was from moderate to almost perfect agreement (see Supplementary Table S2). With an exception of PEA in ultrasound sulcus cartilage grade (53%, 27 of 51 subjects), the PEA ranged from 61% to 82% (31 to 42 of 51 subjects). PCA of original grading ranged from 94% to 98% (48 to 50 of 51 subjects) and modified grading from 69% to 82% (35 to 42 of 51 subjects). In the inter-rater reliability assessment of radiographic OARSI grading, apart from lateral femoral osteophytes (κ_w_ = 0.374), substantial agreements were reached for all measures between two readers (see Supplementary Table S3). The PEA was lowest for medial tibial osteophytes (59%, 47 of 80 subjects) otherwise ranging between 66% and 79% (53 to 63 of 80 subjects). PCA ranged between 95% and 100% (76 to 80 of 80 subjects).

### Diagnostic performance

The AUC, sensitivity, specificity, accuracy, positive predictive value (PPV) and negative predictive value (NPV) quantifying diagnostic accuracy of ultrasound at cutoff grade 1 in all subject population are summarized in Table 2. Regarding ROC analysis, the diagnostic performance of ultrasound to detect any osteophytes in medial and lateral femur and tibia was excellent to good. The ability of ultrasound to identify medial cartilage damage was fair and good in comparison to MOAKS femoral cartilage I and II, respectively. For the lateral femoral condyle the capability was poor and fair, respectively. Ultrasound was able to identify medial meniscal extrusion with excellent and lateral extrusion with good accuracy. Regarding sensitivity, specificity and accuracy, ultrasound reached good to excellent values in detection of most of the morphological abnormalities defined by cutoff grade 1. Low sensitivity, specificity and accuracy values were found for assessment of lateral femoral cartilage I and II, medial femoral cartilage II and medial and lateral meniscal extrusion, and medial femoral cartilage II and lateral meniscal extrusion (Table 2).

The AUC, sensitivity, specificity, accuracy, PPV and NPV quantifying diagnostic accuracy of ultrasound and radiographic OARSI classification at cutoff grade 1 in symptomatic group are summarized in Table 3. Ultrasound performed significantly better than radiography in the detection of medial and lateral femoral osteophytes (*P* < 0.001) and medial meniscal extrusion (*P* = 0.003) with excellent and good AUC values while for radiography ranged from fair to good. Good performance of both modalities to detect osteophytes in medial and lateral tibial bone margins as well as medial cartilage degeneration was observed.

## Discussion

Our study demonstrated that osteophytes, medial meniscal extrusion and morphological articular cartilage changes in the medial femoral condyle of the knee joint can be reliably identified by ultrasound. As recently reported by Riecke *et al.* our results confirmed the ability of ultrasound to discern periarticular bone changes in knee OA^13^. Moreover, we showed that ultrasound is able to detect osteophytes with higher or comparable accuracy than traditional conventional radiography using MRI as the reference. Our findings are also supported by a study of Koski *et al.* who demonstrated that semi-quantitative ultrasound is more sensitive than radiography in the identification of osteophytes in the medial compartment of the knee joint^15^.

The additional diagnostic value of ultrasound over radiography is emphasized by its ability to directly visualize structural changes in cartilage and meniscus^9^,^10^,^11^. Early identification of meniscal extrusion by ultrasound could be especially important since it has been suggested that articular cartilage loss often occurs secondary to meniscal extrusion in patients with early knee OA^10^,^34^. Secondly, three-dimensional and dynamic assessment by ultrasound might help in more precise identification and localization of tissue damage. As it has been already suggested^35^, a combination of imaging modalities is needed in order to identify all aspects involved in knee OA, especially when a source of pain is not evident. Ultrasound could, therefore, reveal early morphological OA changes of individual features when only doubtful minor radiographic degeneration is present, which is known to be a strong predictor for knee OA^36^. For these reasons, our results provide strong evidence that ultrasound has a potential in clinical assessment of knee OA, *e.g.*, as a complementary tool for a clinician who first meets a patient.

In a recent study, the diagnostic performance of semi-quantitative ultrasound for articular cartilage degenerative changes using arthroscopy as the reference was investigated^14^. It was found that positive findings in ultrasound are a strong indicator of arthroscopic cartilage changes but negative findings do not rule out degeneration^14^. Apart from lateral cartilage in all subjects, we found similar relations in our study when determining any cartilage morphological change by ultrasound using MRI as the reference. On the other hand, negative findings in ultrasound confirmed negative full thickness loss determined from MRI, and PPV of full thickness loss by ultrasound was low [40.9% (26.9% to 56.4%) for all subjects and 52.6% (35.5% to 69.2%) for symptomatic subjects] when using grade 1 as a threshold. When using grade 3 as cutoff PPV increased to 83.3% (68.8% to 91.9%) for all subjects and 93.3% (78.7% to 98.2%) for symptomatic subjects showing that ultrasound can well identify cartilage full thickness loss. The possible bias to recognize any cartilage morphological change by ultrasound might have been caused by restriction of the subject to sufficiently flex his/her knee due to advanced OA and thus preventing the depiction of the entire central cartilage. Another limitation is patellar shadowing, which inhibits the ultrasound beam to reach the intra-articular cartilage regions being even more pronounced in the lateral condyle.

In addition to meniscal extrusion, radiographic JSN represents a composite of femoral and tibial cartilage thinning. On the other hand, ultrasound is limited to the assessment of femoral cartilage only. It is still notable that ultrasound performed equally well to radiography in the detection of combined femoral and tibial morphologic changes, thus, supporting earlier findings that cartilage volume and its longitudinal changes in both condyles are strongly related^14^,^37^,^38^.

Nogueira-Barbosa *et al.*^17^. recently reported excellent performance of quantitative and semi-quantitative ultrasound assessment of medial meniscal extrusion defined by 2 mm threshold in patients with chronic knee pain in comparison to MRI. We observed a similar sensitivity for ultrasound to detect meniscal extrusion using a threshold of 3 mm, however, specificity and accuracy were lower than described previously^17^. The differences might be caused by distinct measurement setup as standardized measurement method of meniscal extrusion by ultrasound has not yet been established.

There are several limitations that need mentioning. First, we did not obtain radiographs of asymptomatic subjects for ethical reasons. Consequently, the study sample for cross-comparison with radiography was limited. Secondly, we did not assess meniscal extrusion while the subject was standing, whereas JSN was evaluated from weight-bearing radiographs enhancing the meniscus displacement^16^. Third, the grading system of each imaging modality differs in the definition of cartilage structural change as per se. Semi-quantitative ultrasound quantifies the progression in local cartilage thinning, whereas MOAKS femoral cartilage I describes the area of cartilage degeneration including any cartilage thickness loss and MOAKS femoral cartilage II defines the area of already completely lost cartilage. Last, the subject groups differed in weight/BMI, however, we believe that the assessment of superficial knee structures was not negatively affected by varying thickness of subcutaneous fat layer.

There are some practical limitations, which could be faced when employing ultrasound into clinical practice. Although ultrasound is cheap and widely available its use might be time consuming for the already busy clinician. Additionally, applications differ markedly between the USA where ultrasound is often being performed by sonographers and Europe where radiologists or rheumatologists are performing the examination. On the other hand, obtaining immediate real-time imaging information might have a cost-saving effect, speed up the diagnostic process and make it attractive for clinicians to learn and use.

In conclusion, semi-quantitative ultrasound assessment of the knee joint is an accurate imaging method for detection of tibio-femoral osteophytes, medial meniscal extrusion and medial femoral articular cartilage morphological degeneration in patients with knee OA. Ultrasound is superior to conventional radiography in the detection of femoral osteophytes and medial meniscal extrusion and is able to directly discern femoral cartilage morphological changes and meniscal extrusion. Knee ultrasound could be employed as a complementary imaging technique to radiography, especially when MRI is not justified, to possibly clarify tissue-specific structural OA degeneration not depicted by radiographs.

## Additional Information

**How to cite this article**: Podlipská, J. *et al.* Comparison of Diagnostic Performance of Semi-Quantitative Knee Ultrasound and Knee Radiography with MRI: Oulu Knee Osteoarthritis Study. *Sci. Rep.* **6**, 22365; doi: 10.1038/srep22365 (2016).

## Supplementary Material

Supplementary Information

## Figures and Tables

**Table 1:** Characteristics of symptomatic and asymptomatic subjects.

Characteristic	Symptomatic (*n* = 79)	Asymptomatic (*n* = 80)	*P-value*	All subjects (*n* = 159)
Gender			1.000†	
Female, n (%)	49 (62.0)	50 (62.5)		99 (62.3)
Male, n (%)	30 (38.0)	30 (37.5)		60 (37.7)
Age (y)	59.9 ± 7.8 (34–70)	55.6 ± 13.9 (24–70)	0.318¤	57.7 ± 11.4 (24–70)
Female	60.2 ± 7.5 (39–70)	56.3 ± 12.8 (26–70)	0.324¤	58.2 ± 10.6 (26–70)
Male	#59.3 ± 8.3 (34–70)	#54.5 ± 15.8 (24–70)	0.705¤	#56.9 ± 12.7 (24–70)
Height (cm)	168.9 ± 7.7	168.4 ± 9.3	0.655¤	168.7 ± 8.5
Weight (kg)	83.1 ± 14.4	71.0 ± 12.2	<0.001¤	77.0 ± 14.6
BMI (kg/m^2^)	29.1 ± 4.3	24.9 ± 3.1	<0.001*	27.0 ± 4.3
Knee flexion (°)	129.2 ± 8.7	138.3 ± 5.8	<0.001¤	133.8 ± 8.6
KL grade, n (%)§				
0	2 (2.5)			
1	21 (26.6)			
2	19 (24.1)			
3	20 (25.3)			
4	17 (21.5)			

If not indicated otherwise, the values are means ± standard deviations or age ranges in parentheses.

^†^Chi-square test, exact significance.

^¤^Mann-Whitney U test, exact significance.

^*^Unequal variance *t*-test.

^#^No statistical difference between male and female group (Mann-Whitney U test, *P* > 0.05).

^§^Kellgren-Lawrence (KL) grades given at the subject selection process. Radiographs were evaluated by a rheumatologist (JMK, 25 years of experience in musculoskeletal ultrasound and radiography) who was blinded to any patient details.

**Table 2:** The diagnostic performance values with 95% confidence intervals (CI) of semi-quantitative ultrasound (SQUS) grading of knee structural features in reference to corresponding Magnetic Resonance Imaging Osteoarthritis Knee Score (MOAKS) for symptomatic and asymptomatic subjects.

Feature evaluated by SQUS; MOAKS (*n* = 159)	ROC analysis	Sensitivity	Specificity	Accuracy	PPV	NPV
	*AUC* (95% *CI*)	% (95% *CI*)	% (95% *CI*)	% (95% *CI*)	% (95% *CI*)	% (95% *CI*)
Medial compartment						
Femoral osteophyte	0.969**	98.3	87.0	91.2	81.7	98.9
	(0.944–0.995)	(91.0–99.7)	(79.0–92.2)	(85.8–94.7)	(70.0–89.5)	(94.3–99.8)
Tibial osteophyte	0.889**	89.5	78.4	82.4	69.9	93.0
	(0.832–0.946)	(78.9–95.1)	(69.5–85.3)	(75.7–87.5)	(57.0–80.2)	(86.4–96.6)
Cartilage; Femoral cartilage I	0.784**	74.5	70.2	73.0	81.7	60.6
	(0.715–0.854)	(65.3–82.0)	(57.3–80.5)	(65.6–79.3)	(73.1–88.0)	(47.6–72.2)
Cartilage; Femoral cartilage II	0.893**	97.4	54.2	64.8	40.9	98.5
	(0.837–0.950)	(86.8–100)	(45.3–62.8)	(57.1–71.8)	(26.9–56.4)	(94.4–99.6)
Meniscal extrusion	0.918**	93.0	57.8	70.4	55.2	93.7
	(0.866–0.970)	(83.3–97.2)	(48.2–67.0)	(62.9–77.0)	(42.4–67.4)	(87.2–97.0)
Lateral compartment						
Femoral osteophyte	0.963**	97.8	80.7	85.5	66.7	98.9
	(0.930–0.995)	(88.4–99.6)	(72.5–86.9)	(79.2–90.2)	(52.1–78.6)	(94.9–99.8)
Tibial osteophyte	0.895**	86.1	87.9	87.4	72.6	94.4
	(0.829–0.961)	(72.7–93.4)	(80.8–92.7)	(81.4–91.7)	(57.8–83.6)	(88.7–97.4)
Cartilage; Femoral cartilage I	0.676**	44.7	89.3	76.1	63.6	79.4
	(0.576–0.775)	(31.4–58.8)	(82.2–93.8)	(68.9–82.1)	(49.3–75.9)	(71.0–85.8)
Cartilage; Femoral cartilage II	0.706**	57.1	82.8	80.5	24.2	95.2
	(0.544–0.867)	(32.6–78.6)	(75.8–88.0)	(73.7–85.9)	(9.1–50.4)	(90.5–97.7)
Meniscal extrusion	0.867**	100	12.1	17.6	7.1	100
	(0.780–0.953)	(72.3–100)	(7.8–18.3)	(12.5–24.3)	(1.0–37.0)	(97.5–100)

^**^P < 0.001; SQUS – semi-quantitative ultrasound; MOAKS – Magnetic Resonance Imaging Osteoarthritis Knee Score; ROC – Receiver operating characteristics; *AUC* – Area under the curve; PPV – positive predictive value; NPV – negative predictive value; *CI* – confidence interval.

**Table 3:** The diagnostic performance values with 95% confidence intervals (CI) of semi-quantitative ultrasound (SQUS) and of radiographic Osteoarthritis Research Society International (OARSI) grading of knee structural features in reference to corresponding Magnetic Resonance Imaging Osteoarthritis Knee Score (MOAKS) for symptomatic subjects.

Feature evaluated by SQUS; MOAKS (*n* = 79)	ROC analysis	*P-value*	Sensitivity	Specificity	Accuracy	PPV	NPV
	*AUC* (95% *CI*)		% (95% *CI*)	% (95% *CI*)	% (95% *CI*)	% (95% *CI*)	% (95% *CI*)
Medial compartment							
Femoral osteophyte	0.947**	<0.001	98.1	73.1	89.9	88.1	95.0
	(0.902–0.993)		(90.1–99.7)	(53.9–86.3)	(81.3–94.8)	(76.8–94.4)	(79.5–98.9)
Tibial osteophyte	0.868**	0.617	87.8	70.0	81.0	82.7	77.8
	(0.791–0.946)		(75.8–94.3)	(52.1–83.3)	(71.0–88.1)	(69.8–90.8)	(60.3–89.0)
Medial OA (n = 70)							
Cartilage; Femoral cartilage I	0.852**	0.680	88.1	54.6	82.9	91.2	46.2
	(0.758–0.946)		(77.5–94.1)	(28.0–78.7)	(72.4–89.9)	(81.3–96.1)	(21.8–72.5)
Cartilage; Femoral cartilage II	0.895**	0.312	100	32.5	61.4	52.6	100
	(0.822–0.968)		(88.7–100)	(20.1–48.0)	(49.7–72.0)	(35.5–69.2)	(91.2–100)
Cartilage; Femoral-tibial central cartilage I	0.862**	0.655	89.5	53.9	82.9	89.5	53.9
	(0.775–0.950)		(78.9–95.1)	(29.1–76.8)	(72.4–89.9)	(78.9–95.1)	(29.1–76.8)
Cartilage; Femoral-tibial central cartilage II	0.876**	0.744	96.6	29.3	57.1	49.1	92.3
	(0.791–0.962)		(82.8–99.4)	(17.6–44.5)	(45.5–68.1)	(32.1–66.3)	(80.1–97.3)
Meniscal extrusion	0.939**	0.003	95.5	38.5	74.3	72.4	83.3
	(0.886–0.993)		(84.9–98.7)	(22.4–57.5)	(63.0–83.1)	(57.8–83.4)	(65.0–93.1)
Lateral compartment							
Femoral osteophyte	0.937**	<0.001	100	56.8	79.8	72.4	100
	(0.888–0.985)		(91.6–100)	(40.9–71.3)	(69.6–87.1)	(57.5–83.6)	(90.6–100)
Tibial osteophyte	0.890**	0.640	85.4	86.8	86.1	87.5	84.6
	(0.814–0.965)		(71.6–93.1)	(72.8–94.3)	(76.8–92.0)	(74.1–94.5)	(70.1–92.8)
Lateral OA (n = 28)							
Cartilage; Femoral cartilage I	0.672	0.091	56.3	75.0	64.3	75.0	56.3
	(0.471–0.873)		(33.2–76.9)	(46.8–91.1)	(45.8–79.3)	(50.5–89.8)	(30.3–79.2)
Cartilage; Femoral cartilage II	0.697	0.035	71.4	66.7	67.9	41.7	87.5
	(0.467–0.928)		(35.9–91.8)	(45.4–82.8)	(49.3–82.1)	(15.1–74.1)	(67.5–95.9)
Cartilage; Femoral-tibial central cartilage I	0.740#	0.634	57.9	88.9	67.9	91.7	50.0
	(0.553–0.927)		(36.3–76.9)	(56.5–98.0)	(49.3–82.1)	(71.3–98.0)	(22.7–77.4)
Cartilage; Femoral-tibial central cartilage II	0.787#	0.104	77.8	73.7	75. 0	58.3	87.5
	(0.593–0.980)		(45.3–93.7)	(51.2–88.9)	(56.6–87.3)	(28.8–82.9)	(66.2–96.2)
Meniscal extrusion	0.881*	0.401	100	19.0	39.3	29.2	100
	(0.748–1.000)		(64.6–100)	(7.7–40.0)	(23.6–57.6)	(8.5–64.6)	(84.5–100)
**Feature evaluated by OARSI; MOAKS (n = 79)**							
Medial compartment							
Femoral osteophyte	0.803**		66	92.3	74.7	94.6	57.1
	(0.708–0.898)		(52.6–77.3)	(75.9–97.9)	(64.1–83.0)	(85.0–98.2)	(38.4–74.0)
Tibial osteophyte	0.843**		85.7	76.7	82.3	85.7	76.7
	(0.754–0.932)		(73.3–92.9)	(59.1–88.2)	(72.4–89.1)	(73.3–92.9)	(59.1–88.2)
Medial OA (n = 70)							
JSN; Femoral cartilage I	0.830*		89.8	54.6	84.3	91.4	50.0
	(0.718–0.942)		(79.5–95.3)	(28.0–78.7)	(74.0–91.0)	(81.5–96.2)	(24.6–75.4)
JSN; Femoral cartilage II	0.852**		96.7	27.5	57.1	50	91.7
	(0.757–0.946)		(83.3–99.4)	(16.1–42.8)	(45.5–68.1)	(33.2–66.9)	(79.1–97.0)
JSN; Femoral-tibial central cartilage I	0.839**		91.2	53.9	84.3	89.7	58.3
	(0.735–0.942)		(81.1–96.2)	(29.1–76.8)	(74.0–91.0)	(79.1–95.2)	(32.8–80.1)
JSN; Femoral-tibial central cartilage II	0.862**		96.6	26.8	55.7	48.3	91.7
	(0.768–0.955)		(82.8–99.4)	(15.7–41.9)	(44.1–66.8)	(31.4–65.6)	(79.3–96.9)
JSN; Meniscal extrusion	0.810**		90.9	30.8	68.6	69.0	66.7
	(0.711–0.909)		(78.8–96.4)	(16.5–50.0)	(57.0–78.2)	(54.3–80.6)	(47.5–81.6)
Lateral compartment							
Femoral osteophyte	0.695*		50.0	86.5	67.1	80.8	60.4
	(0.579–0.811)		(35.5–64.5)	(72.0–94.1)	(56.2–76.5)	(66.5–89.9)	(44.4–74.4)
Tibial osteophyte	0.870**		87.8	79.0	83.5	81.8	85.7
	(0.789–0.950)		(74.5–94.7)	(63.7–88.9)	(73.9–90.1)	(67.5–90.7)	(71.3–93.5)
Lateral OA (n = 28)							
JSN; Femoral cartilage I	0.820*		81.3	75.0	78.6	81.3	75.0
	(0.662–0.979)		(57.0–93.4)	(46.8–91.1)	(60.5–89.8)	(57.0–93.4)	(46.8–91.1)
JSN; Femoral cartilage II	0.861*		85.7	52.4	60.7	37.5	91.7
	(0.655–1.000)		(48.7–97.4)	(32.4–71.7)	(42.4–76.4)	(12.8–71.1)	(72.6–97.9)
JSN; Femoral-tibial central cartilage I	0.787#		73.7	77.8	75.0	87.5	58.3
	(0.613–0.960)		(51.2–88.2)	(45.3–93.7)	(56.6–87.3)	(66.2–96.2)	(28.8–82.9)
JSN; Femoral-tibial central cartilage II	0.918**		100	63.2	75.0	56.3	100
	(0.817–1.000)		(70.1–100)	(41.0–80.9)	(56.6–87.3)	(27.2–81.6)	(83.2–100)
JSN; Meniscal extrusion	0.939*		100	57.1	67.9	43.8	100
	(0.839–1.000)		(64.6–100)	(36.6–75.5)	(49.3–82.1)	(16.4–75.6)	(84.5–100)

^**^P < 0.001; ^*^P < 0.01; ^#^*P* < 0.05; *P-value* in ROC analysis determines differences between AUC of SQUS and OARSI grading; SQUS – semi-quantitative ultrasound; OARSI – Osteoarthritis Research Society International; MOAKS – Magnetic Resonance Imaging Osteoarthritis Knee Score; OA – osteoarthritis; ROC – Receiver operating characteristics; *AUC* – Area under the curve; PPV – positive predictive value; NPV – negative predictive value; *CI* – confidence interval.
