# Erratum: Comparison of Diagnostic Performance of Semi-Quantitative Knee Ultrasound and Knee Radiography with MRI: Oulu Knee Osteoarthritis Study

**DOI:** 10.1038/srep33109

**Published:** 2016-09-16

**Authors:** Jana Podlipská, Ali Guermazi, Petri Lehenkari, Jaakko Niinimäki, Frank W. Roemer, Jari P. Arokoski, Päivi Kaukinen, Esa Liukkonen, Eveliina Lammentausta, Miika T. Nieminen, Osmo Tervonen, Juhani M. Koski, Simo Saarakkala

Scientific Reports 6: Article number: 22365; 10.1038/srep22365 published online: 03 01 2016; updated: 09 16 2016.

In the PDF version of this Article, the first paragraph in the Discussion section is incomplete.

“Our study demonstrated that osteophytes, medial meniscal extrusion and morphological articular cartilage changes in the medial femoral condyle of the knee joint can be reliably identified by ultrasound. As recently accuracy than traditional conventional radiography using MRI as the reference. Our findings are also supported by a study of Koski *et al*. who demonstrated that semi-quantitative ultrasound is more sensitive than radiography in the identification of osteophytes in the medial compartment of the knee joint^15^”.

should read:

“Our study demonstrated that osteophytes, medial meniscal extrusion and morphological articular cartilage changes in the medial femoral condyle of the knee joint can be reliably identified by ultrasound. As recently reported by Riecke *et al*. our results confirmed the ability of ultrasound to discern periarticular bone changes in knee OA^13^. Moreover, we showed that ultrasound is able to detect osteophytes with higher or comparable accuracy than traditional conventional radiography using MRI as the reference. Our findings are also supported by a study of Koski *et al*. who demonstrated that semi-quantitative ultrasound is more sensitive than radiography in the identification of osteophytes in the medial compartment of the knee joint^15^”.

