# Supplementary material for: Comparison of Diagnostic Performance of Semi-Quantitative Knee Ultrasound and Knee Radiography with MRI: Oulu Knee Osteoarthritis Study
Source: Sci Rep. 2016 Mar 1;6:22365. doi: 10.1038/srep22365 (PMC4772126; doi:10.1038/srep22365)
Supplement: Supplementary Information [file srep22365-s1.pdf]

Comparison of Diagnostic Performance of Semi-Quantitative Knee Ultrasound and Knee Radiography with MRI: Oulu Knee Osteoarthritis Study

Podlipská, Jana; Guermazi, Ali; Lehenkari, Petri; Niinimäki, Jaakko; Roemer, Frank W; Arokoski, Jari P; Kaukinen, Päivi; Liukkonen, Esa; Lammentausta, Eveliina; Nieminen, Miika T; Tervonen, Osmo; Koski, Juhani M; Saarakkala, Simo

Supplementary Figure S1: Longitudinal ultrasound B-mode images of medial and lateral meniscal extrusion

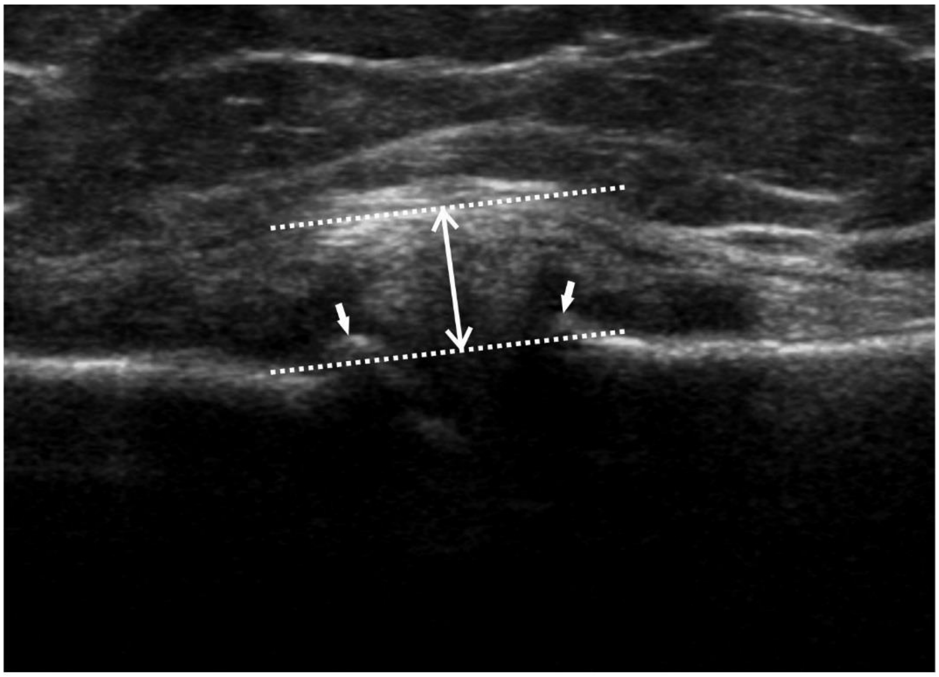

a.

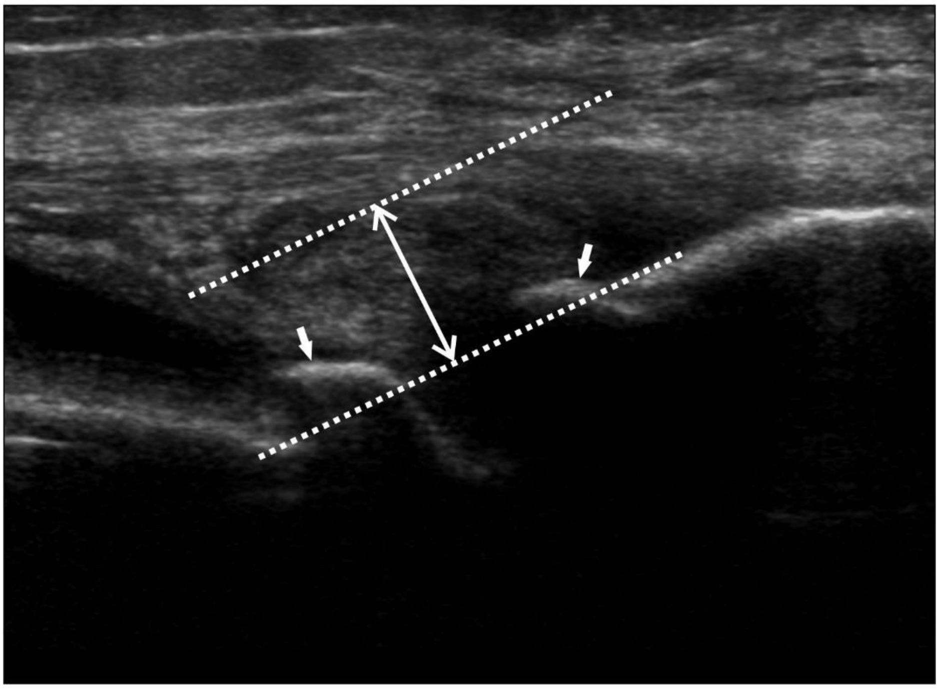

b.

(a) medial meniscal extrusion in 66-year-old symptomatic woman and (b) lateral meniscal extrusion in 55-year-old symptomatic man. The double headed arrow demonstrates the extrusion measurement perpendicular to the dashed line connecting femoral and tibial bone margins. Note the presence of osteophytes (white arrows).

Supplementary Table S1: MRI parameters

Imaging Plane	Sequence Type	TR (ms)/TE (ms)	ETL	Slice thickness (mm)	Field of View (mm)	Acquisition Matrix	No. of Slices	Slice Spacing (mm)	Acquisition Time (min.)
Sagittal	T2-weighted DESS	14.1/5	2	0.6	150 × 150	256 × 256	160		3:16
Sagittal	3D PD-weighted SPACE FS TSE	1200/26	49	0.6	160 × 160	256 × 256	176		8:48
Coronal	PD-weighted TSE	2800/33	4	3	140 × 140	384 × 384	35	3.3	4:09
Coronal	T1-weighted TSE	650/18	2	3	130 × 130	320 × 320	25	3.3	1:56

TR – repetition time, TE – echo time, ETL – echo train length, SE – spin-echo, DESS – dual-echo steady-state, 3D – three dimensional, PD – proton-density, SPACE – Sampling perfection with Application optimized Contrasts using different flip angle Evolution, FS – fat suppressed, TSE – turbo spin-echo

Supplementary Table S2: Intra-rater reliability for reading of semi-quantitative ultrasound features in 51 subjects

Ultrasound feature	Weighted kappa (95% CI)	PEA (%)	PCA (%)	ICC (95% CI)
Medial cartilage[§]	0.679 (0.521–0.838)	70.59	96.08	
Sulcus cartilage[§]	0.519 (0.361–0.677)	52.94	94.12	
Lateral cartilage[§]	0.509 (0.328–0.690)	60.78	96.08	
Medial cartilage[#]	0.718 (0.537–0.899)	80.39	82.35	
Sulcus cartilage[#]	0.462 (0.276–0.648)	62.75	68.63	
Lateral cartilage[#]	***0.453 (0.216–0.690)***	***74.51***	***78.43***	
Medial cartilage[¥]	***0.674 (0.505–0.843)***	***74.51***	***78.43***	
Lateral femoral osteophyte	***0.816 (0.704–0.929)***	***82.35***	***98.04***	
Lateral tibial osteophyte	***0.718 (0.561–0.875)***	***82.35***	***98.04***	
Medial femoral osteophyte	***0.771 (0.609–0.933)***	***82.35***	***98.04***	
Medial tibial osteophyte	***0.756 (0.607–0.905)***	***82.35***	***98.04***	
Medial meniscal extrusion				***0.908 (0.845–0.947)***
Lateral meniscal extrusion				***0.717 (0.552–0.828)***

CI – confidence interval

PEA – percentage of exact agreement

PCA – percentage of close agreement

ICC – intra-class correlation coefficient

[§]Original ultrasound cartilage grading

[#]Modified original ultrasound cartilage grading – grade 0 and 1 combined into grade 0

[¥]Ultrasound medial cartilage grade established as maximum of medial cartilage[#] and sulcus cartilage[#] grades

Note - features applied in the present study are in bold italic font

Supplementary Table S3: Inter-rater reliability for reading of OARSI radiographic features in 80 subjects

OARSI feature	Weighted kappa (95% CI)	PEA (%)	PCA (%)
Medial femoral osteophytes	0.632 (0.490–0.775)	71.25	95.00
Medial tibial osteophytes	0.543 (0.410–0.677)	58.75	97.50
Lateral femoral osteophytes	0.374 (0.214–0.535)	66.25	98.75
Lateral tibial osteophytes	0.568 (0.421–0.715)	67.50	96.25
Medial JSN	0.700 (0.595–0.806)	66.25	100
Lateral JSN	0.647 (0.473–0.820)	78.75	100

OARSI – Osteoarthritis Research Society International

CI – confidence interval

PEA – percentage of exact agreement

PCA – percentage of close agreement

JSN – joint space narrowing
